# Systemic Immune-Inflammation Index (SII) Predicts Poor Survival in Pancreatic Cancer Patients Undergoing Resection

**DOI:** 10.1007/s11605-019-04187-z

**Published:** 2019-03-28

**Authors:** Gerd Jomrich, Elisabeth S. Gruber, Daniel Winkler, Marlene Hollenstein, Michael Gnant, Klaus Sahora, Martin Schindl

**Affiliations:** 1 Department of Surgery, Comprehensive Cancer Center (CCC), Medical University Vienna and Pancreatic Cancer Unit, Spitalgasse 23, 1090 Vienna, Austria; 2 Vienna University of Economics and Business, Welthandelsplatz 1, AD 1020 Vienna, Austria

**Keywords:** Pancreatic ductal adenocarcinoma, Systemic immune-inflammation index, Inflammation

## Abstract

**Background:**

The systemic immune-inflammation index based on peripheral neutrophil, lymphocyte, and platelet counts has shown a prognostic impact in several malignancies. The aim of this study was to determine the prognostic role of systemic immune-inflammation index in patients with pancreatic ductal adenocarcinoma undergoing resection.

**Methods:**

Consecutive patients who underwent surgical resection at the department of surgery at the Medical University of Vienna between 1995 and 2014 were included into this study. The systemic immune-inflammation index was calculated by the formula platelet*neutrophil/lymphocyte. Optimal cutoffs were determined using Youden’s index. Uni- and multivariate analyses were calculated by the Cox proportional hazard regression model for overall survival.

**Results:**

Three hundred twenty-one patients were included in this study. Clinical data was achieved from a prospective patient database. In univariate survival analysis, elevated systemic immune-inflammation index was found to be significantly associated with shortened patients’ overall survival (*p* = 0.007). In multivariate survival analysis, systemic immune-inflammation index remained an independent prognostic factor for overall survival (*p* = 0.004). No statistical significance could be found for platelet to lymphocyte ratio and neutrophil to lymphocyte ratio in multivariate analysis. Furthermore, area under the curve analysis showed a higher prognostic significance for systemic immune-inflammation index, compared to platelet to lymphocyte ratio and neutrophil to lymphocyte ratio.

**Conclusion:**

A high systemic immune-inflammation index is an independent, preoperative available prognostic factor in patients with resectable pancreatic ductal adenocarcinoma and is superior to platelet to lymphocyte ratio and neutrophil to lymphocyte ratio for predicting overall survival in pancreatic ductal adenocarcinoma patients.

## Introduction

Pancreatic ductal adenocarcinoma (PDAC) is the ninth most common malignancy and ranks fifth place of cancer-related death in western countries with inclining incidence.^1^ Despite the development of multimodal approaches, combining surgical resection with perioperative chemo-(radio)therapy, 5-year survival rate for patients diagnosed with PADC remains poor with less than 5%.^2^

Inflammation, as one of the hallmarks of cancer, is an acknowledged factor in tumor biology.^3,4^ Inflammation-driven tumorigenesis and tumor progression plays a crucial role in malignant diseases.^3,5^ Systemic inflammatory response (SIR) in the context of tumor-associated inflammation has been demonstrated to diminish outcome and be of major prognostic importance in various cancers.^6,7^ A number of promising and potentially prognostic immunologic and histologic biomarkers have been investigated in PDAC.^8,9^ However, evaluation of these biomarkers is often expensive and time-consuming. Thus, investigation of tumor-driving inflammation-based components is of major importance and targeting pathways of inflammatory response might become a cornerstone of cancer treatment.^10^ In PDAC, outcome prediction mainly depends on clinical and pathological factors, such as tumor size, lymph node involvement, and distant metastases as well as resection margin.^11^ Notably, these factors are obtained post-operatively through evaluation of the surgical specimen and current available prognostic markers do not allow to preoperatively predict outcome. Therefore, identification of easy-available markers might help to determine individual treatment approaches. The utility of inflammation-based scores, such as neutrophil-lymphocyte ratio (NLR), platelet-lymphocyte ratio (PLR) and systemic immune-inflammation index (SII), are based on routinely obtained markers that are available before surgery. Elevated SII has been reported to be associated with clinico-pathological parameters and has been proven to be an independent prognostic factor in a number of malignancies, including PDAC.^12–16^ However, no data exists until now, describing the prognostic value of the SII in PDAC after neoadjuvant treatment. The aim of the present study was to assess the prognostic value of SII in patients undergoing a potentially curative resection for PDAC with or without neoadjuvant therapy. Furthermore, the SII is compared with NLR and PLR in predicting survival in this cohort of patients.

## Material and Methods

Patients undergoing resection for PDAC between 1995 and 2014 at the Department of Surgery, Medical University of Vienna, were identified from a prospectively maintained PDAC database. Both patients who primarily underwent surgery and patients with borderline resectable disease according to NCCN guidelines who were treated by chemotherapy or radio-chemotherapy before resection were included.^17^ The study was approved by the Ethics Committee of the Medical University Vienna, Austria, in accordance with the Helsinki declaration (EK 1166/2013). Clinico-pathological data were assembled from medical records, including, gender, age, preoperative neutrophil, lymphocyte and platelet counts, tumor site, histopathological tumor grading, staging (TNM) according to the 8th edition of the Union for International Cancer Control (UICC)/American Joint Committee on Cancer (AJCC), neoadjuvant treatment and surgical resection technique. Tumor resection margin status (*R*) was classified as R0 or R1 (1-mm tumor-free margin). Patients with distant metastases at time of diagnosis and death from other cause within 30 days post-surgery as well as patients who had recently pyrexia (axillary ≥ 37.2 °C/99.0 °F), any form of active infection or chronic inflammatory disease were excluded from the study. Each patient was discussed in the multidisciplinary team meeting before surgery. Neo-/adjuvant chemotherapy was administered according to the standard regimens available at the respective period. For neoadjuvant treatment, 5-fluorouracil was used from 1995 to 1998, whereas gemcitabine-based regimens (gemcitabine monotherapy and combinations with oxaliplatin, erlotinib, and nab-Paclitaxel) or FOLFIRINOX were used from 1999 onwards. The present standard for neoadjuvant treatment since several years consists of either FOLFIRINOX or Gem/nab-Pac depending on patients’ condition. For adjuvant chemotherapy, 5-fluorouracil-based regimens were used between 1995 and 1998 and gemcitabine-based regimens were administered thereafter. All patients were regularly followed thereafter with physical examination, tumor marker, and computed tomographic scan every 3 months for the first 2 years and every 6 months until 5 years after surgery.

Blood samples were obtained within 7 days prior to surgery. NLR, PLR, and SII were calculated as previously described: NLR = neutrophils / lymphocytes, PLR = platelet / lymphocytes, and SII = platelets × neutrophils / lymphocytes.^16^

### Statistical Analysis

Statistical analysis was performed using the R statistical software (Version 3.4.3) with the “Survival,” “pROC,” and “Optimal Cutpoints” packages.^18–21^ To evaluate the discriminatory ability of the SII, NLR, and PLR, ROC curves were generated and the area under the ROC curves (AUROCs) was measured and compared. For bivariate analysis, to investigate relationships between SII, NLR, and PLR and clinico-pathological parameters, *t* test and the Wilcoxon test were used as appropriate. Univariate and multivariate analyses were conducted using the Cox proportional hazard model. In the multivariable model, SII, NLR, and PLR could not be included into together, due to multicollinearity. Therefore, the stepwise regression analysis for multivariate Cox models, SII, NLR, and PLR could not be included into the model together due to multicollinearity. Discrimination ability was compared using the receiver operating curve. Optimal cutoff values for SII, NLR, and PLR were determined using Youden’s index, which maximizes the sum of sensitivity and specificity. Graphically, it is represented by the distance between the 45-degree line and the ROC^22,23^. The graphical analysis was performed using the Kaplan-Meier survival curve estimator and analyzed by the log-rank test. Overall survival (OS) was defined as time between primary surgery and the patients’ death. Death from other cause than PDAC or survival until the end of the observation period was considered as censored observations. Disease-free survival (DFS) was defined from the day of surgery until first evidence of disease progression. Categorical data was analyzed using the chi-squared test. Continuous data was either analyzed using the *t* test form normally distributed values or the Wilcoxon rank-sum test.

## Results

In the present study, a total of 321 patients (169, 52.2% male) with a median age of 68.5 (range, 35.9–92.3) years were included. The majority of patients (201, 62.6%) presented with a moderately differentiated tumor (G2) and 43 (13.3%) patients received neoadjuvant treatment prior to resection. In 255 (78.7%) cases, the tumor was located in the head of the pancreas and in 238 (73.5%) positive lymph node was found at pathological assessment. In accordance with the 8th edition UICC/AJCC classification, 265 (82.6%) patients showed stage II disease and 153 (47.2%) patients underwent biliary drainage preoperatively. Clinico-pathological data are presented in Table 1.

**Table 1:** Association of the SII with clinico-pathological parameters in pancreatic ductal adenocarcinoma

Factors	All		SII		
			High (≥ 873)	Low (< 873)	*p* value
*n*	324		120	204	
Age, mean (SD)	68.25	(9.74)	68.81 (9.21)	67.91 (10.04)	> 0.1(*)
Sex					> 0.1
Male	169	52.16%	65	104	
Female	155	47.84%	55	100	
(y)pT					> 0.1
0	2	0.62%	1	1	
1	24	7.41%	5	19	
2	47	14.51%	18	29	
3	234	72.22%	87	147	
4	17	5.25%	9	8	
(y)pN					> 0.1
0	86	26.54%	27	59	
1	238	73.46%	93	145	
(y)G					> 0.1
1	15	4.63%	4	11	
2	204	62.96%	72	132	
3	105	32.41%	44	61	
*R*					0.027
0	254	78.40%	86	168	
1	70	21.60%	34	36	
UICC staging					> 0.1
I	41	12.65%	15	26	
II	265	81.79%	96	169	
III	18	5.56%	10	8	
Neoadjuvant treatment					> 0.1
yes	43	13.27%	14	29	
no	281	86.73%	106	175	
Jaundice					0.041
No	163	50.31%	51	112	
Yes	161	49.69%	69	92	
CA 19-9					0.013
Unknown	24	7.41%	9	15	
≤ 114 kU/L	143	44.14%	42	101	
> 114 kU/L	157	48.46%	69	88	
Lymph node-ratio					> 0.1
Unknown	23	7.10%	7	16	
≤ 0.2	196	60.49%	71	125	
> 0.2	105	32.41%	42	63	
Nicotine					> 0.1
Unknown	3	0.93%	1	2	
Yes	135	41.67%	55	80	
No	186	57.41%	64	122	
Pain					> 0.1
Yes	130	40.12%	45	85	
No	194	59.88%	75	119	
Pancreatitis					> 0.1
Yes	63	19.44%	20	43	
No	261	80.56%	100	161	
Diabetes					> 0.1
Unknown	12	3.70%	3	9	
Yes	73	22.53%	29	44	
No	239	73.77%	88	151	
Stent					> 0.1
Yes	153	47.22%	60	93	
No	171	52.78%	60	111	
Surgical procedure					0.0345
PPPD	181	55.86%	65	116	
Whipple	71	21.91%	35	36	
Distal resection	60	18.52%	15	45	
Total pancreatectomy	12	3.70%	5	7	
Localization					> 0.1
1	255	78.70%	100	155	
2	24	7.41%	8	16	
3	45	13.89%	12	33	

(*) Using *t* test

SD, standard deviation; UICC, Union for International Cancer Control; SII, systemic immune-inflammation index

**Fig. 1:**
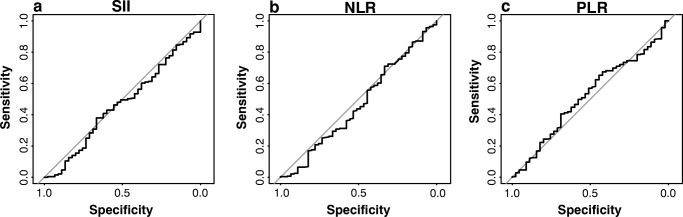
Receiver operating characteristic (ROC) curves were generated to evaluate the discriminatory ability of the SII (**a**), NLR (**b**), and PLR (**c**). The area under the ROC curves (AUROCs) were measured and compared

The median OS was 18.5 months (range, 1.5–198 months) and the rate of 3- and 5-year OS was 25.63% and 8.44%, respectively. The optimal cutoff values for SII, PLR, and NLR were 873, 179, and 225, respectively. With the defined cutoffs, 119 patients had SII ≥ 873, 125 patients had PLR ≥ 179, and 225 patients NLR ≥ 2.15 before surgery. Using bivariate analysis, significant relationship between elevated SII and clinico-pathological parameters was found for resection margin (*p* = 0.03), jaundice (*p* = 0.04), CA 19–9 (*p* = 0.01), and surgical procedure (*p* = 0.04) (Table 1). The median OS for patients with high SII was 14.2 (range, 1.5–128.2) months and 20.5 (range, 1.6–200.8) months for patients with low SII respectively. In the entire cohort, using overall survival as an end-point, the area under the receiver operator curve was 0.46 (CI, 0.37–0.55) for SII, 0.46 (CI, 0.36–0.56) for NLR, and 0.51 (CI, 0.42–0.61) for PLR with no significant difference in discrimination ability between SII, NLR, and PLR regarding OS was found (Fig. 1).

**Fig. 2:**
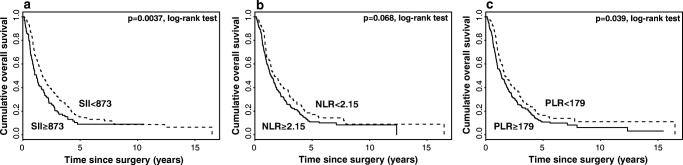
Kaplan-Meier curves for overall survival (OS) stratified by SII (**a**), NLR (**b**) and PLR (**c**)

Kaplan-Meier curve survival analysis for all patients revealed that low SII (*p* = 0.004) and PLR (*p* = 0.04) were significantly associated with longer OS, whereas no significance was found for NLR (Fig. 2).

Univariate Cox proportional hazard regression revealed that SII, PLR, age, jaundice, resection margin, CA 19-9, lymph node-ratio, and tumor size were significantly associated with OS. Similarly, age, jaundice, lymph node-ratio, and tumor size are significantly associated with DFS whereas SII, NLR, and PLR was not (Table 2).

**Table 2:** Univariate Cox regression analysis estimating the influence of the SII, NLR, PLR and clinico-pathological parameters on overall survival and disease-free survival in patients with ductal adenocarcinoma of the pancreas

Overall survival				Disease-free survival		
	RR	CI (95%)	*p* value	RR	CI (95%)	*p* value
SII						
≥ 873 vs. < 873	0.6979	0.5469–0.8907	0.0038	0.8147	0.6288–1.0556	0.1211
NLR						
≥ 2.15 vs. < 2.15	0.7852	0.6054–1.0184	0.0683	0.8536	0.6455–1.1286	0.2666
PLR						
≥ 179 vs. < 179	0.772	0.6031–0.9882	0.0399	0.808	0.6205–1.0522	0.1136
UICC stage						
II vs. I	1.0668	0.7438–1.5301	0.7252	1.1808	0.7898–1.7653	0.4179
III vs. I	1.4071	0.7658–2.5852	0.2712	1.2352	0.6366–2.3964	0.5323
Grading						
2 vs. 1	0.7087	0.4174–1.2032	0.2023	0.6882	0.3973–1.1922	0.1826
3 vs. 1	1.0347	0.5996–1.7855	0.9026	1.0086	0.5719–1.7785	0.9765
Sex						
Male vs. female	1.0843	0.8562–1.373	0.5019	1.0475	0.8145–1.3472	0.7175
Age	0.9881	0.9769–0.9993	0.038	0.9838	0.9714–0.9964	0.0116
Jaundice						
Yes vs. no	1.4243	1.1246–1.8039	0.0033	1.4186	1.1013–1.8272	0.0068
*R*						
1 vs. 0	1.4382	1.0866–1.9037	0.0111	1.3526	0.9989–1.8315	0.0508
CA 19-9						
≥ 114 vs. < 114 kU/L	1.3148	1.0281–1.6815	0.0292	1.2842	0.9899–1.6661	0.0596
Lymph node-ratio						
≥ 0.2 vs. < 0.2	1.8094	1.4006–2.3375	< 0.0001	1.6615	1.264–2.184	0.0003
T-staging						
1 vs. 0	0.2554	0.0592–1.1006	0.067	0.1249	0.0162–0.9622	0.0458
2 vs. 0	0.231	0.0553–0.9647	0.0445	0.1209	0.0162–0.9034	0.0395
3 vs. 0	0.2674	0.0657–1.0876	0.0654	0.1266	0.0173–0.9263	0.0418
4 vs. 0	0.3471	0.0786–1.5319	0.1624	0.1349	0.0172–1.0561	0.0564
N-staging						
1 vs. 0	1.158	0.8836–1.5176	0.2876	1.0838	0.8162–1.4391	0.5781
Pain						
Yes vs. no	0.9929	0.7804–1.2632	0.9535	1.0445	0.8088–1.3488	0.7388
Pancreatitis						
Yes vs. no	0.9514	0.7064–1.2815	0.7431	0.8858	0.6426–1.2212	0.4592
Nicotine						
Yes vs. no	1.106	0.8705–1.406	0.409	1.0901	0.8457–1.4052	0.5053
Diabetes						
Yes vs. no	1.136	0.8545–1.51	0.38	1.1107	0.8214–1.502	0.4953
Neoadjuvant treatment						
Yes vs. no	1.3449	0.9616–1.881	0.0834	1.2416	0.8711–1.7696	0.2314
Stent						
Yes vs. no	1.1175	0.883–1.4142	0.3553	1.1664	0.9076–1.4989	0.2291
Surgical procedure						
PPPD						
Whipple	1.0712	0.7961–1.4414	0.6497	1.0691	0.7808–1.4638	0.6769
Distal resection	0.8498	0.6152–1.1739	0.3236	1.2939	0.6334–2.6432	0.4796
Total pancreatectomy	1.4092	0.7425–2.6747	0.294	0.9609	0.6824–1.353	0.8192
Localization						
2 vs. 1	0.8828	0.5386–1.4469	0.621	1.0087	0.6055–1.6802	0.9736
3 vs. 1	0.9289	0.6605–1.3062	0.6714	1.0415	0.7199–1.5069	0.829

UICC, Union for International Cancer Control; CI, confidence interval; RR, relative risk; Ref., reference; SII, systemic immune-inflammation index; PLR, platelet lymphocyte ratio; NLR, neutrophil lymphocyte ratio; PPPD, pylorus-preserving pancreaticoduodenectomy

Furthermore, no statistical significance was found for OS and DFS in univariate Cox proportional hazard regression for neoadjuvant treatment (*p* = 0.08; RR, 1.35; CI, 95 0.96–1.88; and *p* = 0.23; RR, 1.24; CI, 95 0.87–1.77; respectively; Table 2).

Multivariate Cox-regression analysis using SII as bivariate variable revealed that a high SII (*p* = 0.016; RR, 0.71; CI, 95% 0.54–0.94), positive resection margin (*p* = 0.03; RR, 1.46; CI, 95% 1.05–2.03), and a high lymph node-ratio (*p* < 0.001; RR, 1.77; CI, 95% 1.32–2.36), but not NLR and PLR, are independent risk factors for OS (Table 3). No statistical significant association could be found for SII, NLR, and PLR in the multivariate Cox models for DSF (Table 4).

**Table 3:** Multivariate Cox regression analysis estimating the influence of the SII and clinico-pathological parameters on overall survival in patients with ductal adenocarcinoma of the pancreas

Overall survival	SII		
	RR	CI (95%)	*p* value
SII			
≥ 873 vs. < 873	0.7138	0.5427–0.9388	0.0159
UICC stage			
II vs. I	0.7796	0.493–1.2329	0.2871
III vs. I	0.9423	0.4591–1.9344	0.8714
Grading			
2 vs. 1	0.5881	0.3181–1.0875	0.0906
3 vs. 1	0.8104	0.4285–1.5328	0.5179
Sex			
Male vs. female	0.9328	0.7155–1.2162	0.6074
Age	0.9874	0.9749–1.0001	0.0514
Jaundice			
Yes vs. no	1.1994	0.9094–1.5819	0.1979
*R*			
1 vs. 0	1.4561	1.0457–2.0275	0.0261
CA 19-9			
≥ 114 vs. < 114 kU/L	1.1642	0.8795–1.541	0.288
Lymph node-ratio			
≥ 0.2 vs. < 0.2	1.7687	1.3247–2.3615	< 0.001

UICC, Union for International Cancer Control; CI, confidence interval; RR, relative risk; SII, systemic immune-inflammation index

**Table 4:** Multivariate Cox regression analysis estimating the influence of the SII and clinico-pathological parameters on disease-free survival in patients with ductal adenocarcinoma of the pancreas

Disease-free survival	SII		
	RR	CI (95%)	*p* value
SII			
≥ 873 vs. < 873	0.787	0.5885–1.0524	0.1062
UICC stage			
II vs. I	1.0855	0.6388–1.8446	0.7617
III vs. I	1.0861	0.495–2.3831	0.8367
Grading			
2 vs. 1	0.5279	0.2826–0.9861	0.0451
3 vs. 1	0.7407	0.3878–1.4148	0.3633
Sex			
Male vs. female	0.9176	0.6892–1.2217	0.5562
Age	0.9823	0.9685–0.9964	0.0138
Jaundice			
Yes vs. no	1.2138	0.9041–1.6295	0.1973
*R*			
1 vs. 0	1.405	0.984–2.006	0.0613
CA 19-9			
≥ 114 vs. < 114 kU/L	1.1385	0.8459–1.5323	0.3921
Lymph node-ratio			
≥ 0.2 vs. < 0.2	1.4942	1.0995–2.0306	0.0103

UICC, Union for International Cancer Control; CI, confidence interval; RR, relative risk; SII, systemic immune-inflammation index

## Discussion

Inflammation plays a key role in tumor initiation, malignant conversion, and metastasis and influences the host anti-tumor immunity.^3–5^ The present study investigated the clinical and prognostic value of preoperative SII, NLR, and PLR in patients with PDAC undergoing resection and competed their predictive accuracy. Overall, SII, but not NLR and PLR was an independent prognostic factor for OS in patients with PDAC undergoing resection.

The relationship between tumor and inflammation was first described by Virchow in 1863, and later in 1986 by Dvorak as “Tumors: Wounds that do not heal.” Meanwhile, inflammation is known as one of the hallmarks of cancer.^4,24,25^ Increasing data shows the close relationship between tumorigenesis, tumor progression, and metastasis.^4–6^ The major prognostic impact of inflammatory markers can be ascribed to a cytokine-driven immunogenic tumor microenvironment and a significant prognostic role of inflammation-based biomarkers and scores has recently been shown in a number of malignant diseases.^5,7,26–28^ One of the newly emerging prognostic scores is the SII, based on platelets, neutrophils, and lymphocytes. As a combination of both NLR and PLR, SII firstly has been confirmed as superior prognostic factor in hepatocellular carcinoma and then in small cell lung cancer reflecting patient’s inflammatory status.^15,16^ In a number of malignancies, including PDAC, an elevated preoperative SII plays a key role in prognosis estimation.^12–16,29–31^ This is the first study that has proven the prognostic value of the SII and is superior to PLR.

It has been proposed that SII is able to predict tumor recurrence in a highly inflammatory tumor microenvironment with infiltrating immune cells that promote tumorigenesis and dissemination.^5,32^ Neutrophils activate endothelium and parenchymal cells via secretion of soluble factors that enhance tumor cell adhesion at distant sites.^33–35^ Increasing numbers of blood neutrophils and platelets have been associated with tumor progression and diminished clinical outcome in a number of solid tumors.^36,37^ Lymphocytes inhibit tumor cell proliferation and migration through induction of cytotoxic cell death and thus play a key role in cancer immuno-surveillance.^5^ On the basis of these findings, several inflammation-based scores have emerged as prognostic indicators in cancer patients.

Recently published data is diverging regarding the prognostic value of NLR and PLR. The NLR, combining circulating neutrophil and lymphocyte counts, and the PLR, combining circulating platelets and lymphocyte counts, has been associated with impaired survival in lung and ovarian cancers, while in PDAC results remain inconsistent.^38–42^ Whereas Mowbray et al. found preoperative NLR to be an independent prognostic predictor, Chawla et al. reports that neither NLR nor PLR predicts survival in patients who underwent pancreatectomy for PDAC.^43,44^

A high SII, consisting of high neutrophil and platelet as well as low lymphocyte counts, indicates inflammation activity that may be associated with poor survival through enhanced tumor invasion and metastases. Investigating the prognostic capacity of SII, NLR, and PLR, our results were consistent with those of Chawla et al., revealing that preoperative SII, but not NLR and PLR, is an independent prognostic factor for OS in patients with resectable PDAC.^43^ Recently, Haldar and Ben-Eliyahu critically discussed the impact of perioperative β-adrenergic blockade and COX2 inhibition on cancer outcomes.^45^ Thus, patients with resectable PDAC who have elevated preoperative SII might benefit from anti-inflammatory and/or anti-immunotherapy prior and after surgery.

Even though the results of the present study demonstrate that the SII is an independent prognostic factor in patients with PDAC undergoing resection, it has several limitations. Although the patients were prospectively entered into a database, a retrospective analysis was performed with a selection bias by the availability of complete blood cell count before surgery in daily practice. The cohort represents the experience of one center that needs to be validated by external cohort from another center.

There are no consensual cutoff values for inflammation indices. The majority of studies determine individual cutoff levels by their relevance and significance, showing a significant prediction of survival when applied to the same patients’ cohort. As a result, there is a wide range of cutoff values for these indices. However, the present study demonstrated that the SII provided the strongest survival prediction compared to NLR and PLR in patients with PDAC undergoing surgery. Emphasis should be given to determine significant cutoff levels for inflammatory indices that stay valid when applied to independent cohorts of patients.

The administration of different neo-/adjuvant chemotherapy regimens and changing policies of treating patients with borderline disease during the study period may have influenced the study result. However, it reflects the real-world situation and patients included in the study were treated with standard regimens that were available at the respective period. We did not analyze the differences of prognostic strength of SII during different time intervals reflecting variations in neo-/adjuvant chemotherapy regimens. However, we could proof the prognostic value of SII for the entire observation period, regardless neoadjuvant treatment was administered or not. We admit that in order to draw representative conclusions for the association between inflammatory activity and prognosis in patients undergoing neoadjuvant treatment, a cohort with little variations in neoadjuvant regimens should be analyzed.

High and low SII was equally distributed in most of the items characterizing the study cohort. However, resection rates, CA19-9 and bilirubin blood concentrations were different between patients with high and low inflammatory activity. The difference of resection rates interesting observation that needs to be addressed by further studies as it may represent an important factor for treatment decision. There is evidence that tumor invasion initiates host inflammatory response and one could argue that the extent of tumor cell infiltration into the mesopancreatic compartment both stimulates inflammatory activity and influences the likelihood of complete resection. Similarly, CA19-9, serves as a surrogate marker of tumor burden and thereby associated with inflammation.

## Conclusion

In summary, the present study shows that preoperative SII is an independent predictor of OS in patients with PDAC undergoing pancreatic resection that is superior to NLR and PLR. Measurement of SII is easily applicable and of low cost. Patients with preoperatively elevated SII might benefit from anti-inflammatory and/or anti-immunotherapy.
